# Vision-Based Moving Obstacle Detection and Tracking in Paddy Field Using Improved Yolov3 and Deep SORT

**DOI:** 10.3390/s20154082

**Published:** 2020-07-22

**Authors:** Zhengjun Qiu, Nan Zhao, Lei Zhou, Mengcen Wang, Liangliang Yang, Hui Fang, Yong He, Yufei Liu

**Affiliations:** 1 College of Biosystems Engineering and Food Science, Zhejiang University, Hangzhou 310058, China; zjqiu@zju.edu.cn (Z.Q.); 3150100510@zju.edu.cn (N.Z.); zhoulei_17@zju.edu.cn (L.Z.); hfang@zju.edu.cn (H.F.); yhe@zju.edu.cn (Y.H.); 2 Key Laboratory of Spectroscopy Sensing, Ministry of Agriculture and Rural Affairs, Hangzhou 310058, China; 3 Ministry of Agriculture Key Laboratory of Molecular Biology of Crop Pathogens and Insects, Institute of Pesticide and Environmental Toxicology, Zhejiang University, Hangzhou 310058, China; wmctz@zju.edu.cn; 4 Faculty of Engineering, Kitami Institute of Technology, Koen-cho 165, Kitami, Hokkaido 090-8507, Japan; yang@mail.kitami-it.ac.jp

**Keywords:** machine vision, deep learning, detecting and tracking, moving obstacles, paddy field

## Abstract

Using intelligent agricultural machines in paddy fields has received great attention. An obstacle avoidance system is required with the development of agricultural machines. In order to make the machines more intelligent, detecting and tracking obstacles, especially the moving obstacles in paddy fields, is the basis of obstacle avoidance. To achieve this goal, a red, green and blue (RGB) camera and a computer were used to build a machine vision system, mounted on a transplanter. A method that combined the improved You Only Look Once version 3 (Yolov3) and deep Simple Online and Realtime Tracking (deep SORT) was used to detect and track typical moving obstacles, and figure out the center point positions of the obstacles in paddy fields. The improved Yolov3 has 23 residual blocks and upsamples only once, and has new loss calculation functions. Results showed that the improved Yolov3 obtained mean intersection over union (mIoU) score of 0.779 and was 27.3% faster in processing speed than standard Yolov3 on a self-created test dataset of moving obstacles (human and water buffalo) in paddy fields. An acceptable performance for detecting and tracking could be obtained in a real paddy field test with an average processing speed of 5–7 frames per second (FPS), which satisfies actual work demands. In future research, the proposed system could support the intelligent agriculture machines more flexible in autonomous navigation.

## 1. Introduction

The increase in global population has resulted in an increasing demand for crops [1]. Paddy fields are one of the main production fields of crops, where lots of intense farming work is conducted such as seedling cultivation, transplanting, fertilization, weeding, pest control and harvesting [2]. Although the human population has been increasing, the ratio of labor force working in agriculture constantly declines [3], which has led smart agriculture to be an acceptable approach to meet the ever-increasing demand for agricultural food. Developing intelligent agriculture machines, which has autonomous navigation system technology is an important branch in the process of smart agriculture [4]. It can effectively enhance the quality and efficiency of field operations, improve the accuracy of farm work, and reduce labor intensity. Robotics is a universal approach to objectify intelligent agricultural machines [5]. With the advantages of robotics, many research projects were conducted over the years trying to achieve robotization in paddy fields to reduce human labor. In earlier stages of robotization, using various positioning systems to complete path planning drew a lot of attention [6]. The positioning system mounted on agriculture machine could generate the location in real time to support the intelligent machine to complete path planning and control assignments. [7]. 

Nagasaka et al. [8] combined a real-time kinematic global positioning system (RTK-GPS) and fiber optic gyroscope (FOG) sensors to develop an automated six-row rice transplanter whose root mean square (RMS) deviation from the desired straight path was approximately 5.5 cm at 0.7 km/s. Liu et al. [2] developed an unmanned airboat with an unmanned aerial vehicle (UAV) system for navigation in paddy field, and the RMS lateral errors were 0.17 m, 0.10 m, 0.11 m in 3 predefined paths. Gonzalez-de-Santos P et al. [9] developed a system consisting of unmanned ground vehicle (UGV) and UAV with sprayer for weeding and pest control which met the requirements of practical application. Zhang et al. [10] proposed and developed a robot combine harvester which could be applied in automatic paddy harvesting in Hokkaido. Those researchers focused on path planning in a structured paddy field environment without obstacles. However, how to overcome the complicated water environment and avoid unknown obstacles are still challenges the intelligent machine working in paddy field faces. In practical applications, to avoid obstacles, especially living obstacles such as farmers and livestock it is necessary to have intelligent agriculture machines in paddy fields. As a matter of fact, agriculture machines without avoidance function caused a high accident rate in the past. According to a survey of the National Institute for Occupational Safety and Health (NIOSH) of the United States, in the year 2016, 417 farmers died from a work-related injury mainly caused by transportation incidents. Meanwhile, about 100 agricultural workers suffered lost-work time injuries every day [11]. A national survey of farm accidents conducted by the Teagasc National Farm Survey (NFS) in 2018 showed that farm accidents had risen by 13% in the last five years and by 31% in the last 10 years [12]. Moreover, humans, other moving obstacles like water buffaloes also need to be concerned. Water buffaloes still are the common animal power in paddy field in developing countries of South Asia. 

In order to enhance the stability and universality of intelligent agriculture machines for working in unstructured open paddy fields and make path planning more flexible which could greatly help reduce the accident rate in paddy fields, detecting and tracking the moving obstacles like humans in paddy fields are necessary. Currently, machine vision and deep-learning technologies are widely used in agriculture fields [13,14]. With the rapid development of machine vision and deep-learning technologies, researchers in this stage start to equip those intelligent agricultural machines with image acquisition device [15], and adopt image processing approach to assist positioning system [16] to plan the path as well as avoid obstacles. 

Zhou et al. [17] handled two frame images obtained by a red, green and blue (RGB) camera from agricultural mobile robot with Harris feature points extracting and matching, bilinear model, least square optimization method, matrix transformation to detect moving obstacles in the environment. Pajares G et al. [18] designed an obstacle detection method by analyzing images on the $b^{*}$ channel in the CIELabcolor space. Simultaneously, binary images were also obtained to get the texture information for each pixel. With the rapid development of deep learning, the images processing ability has been significantly improved [19] which has greatly assisted the applications of object detection and classification in agriculture. Liu et al. [20] developed a pipeline for localization and classification of paddy field pests using a saliency map and deep convolutional neural network (DCNN) which achieved a mean accuracy precision (mAP) of 0.951. Christiansen et al. [21] combined deep learning and anomaly detection to develop an algorithm named DeepAnomaly, which was applied in agricultural field to detect obstacles. The algorithm was 7.28-times faster than regions convolutional neural network (RCNN) in processing per image with high accuracy in detecting people at the range of 45–90 m. 

From the above, adopting an image processing approach combined with positioning system to support development of intelligent agriculture machines has become a new main trend, in which avoiding obstacles is an interesting and attractive point. With an image processing approach and positioning system, intelligent agriculture machines could handle some emergencies like avoiding obstacles instead of just following planned paths. To avoid obstacles, the machines have to be clear where those obstacles are. In this paper, the authors attempted to develop a machine vision system with deep-learning method to detect, recognize and track the moving obstacles in paddy field. The developed machine vision system was easy to mount on agricultural machines. The proposed system could provide real-time identification and classification of obstacles for agricultural machines in an open, unstructured paddy field environment. This research could improve the robustness of obstacle avoidance, and benefit the development of intelligent agricultural machines. 

## 2. Materials and Methods

### 2.1. Sensors and System Components

In this research, an industrial camera (DFK-23U445, IMAGING SOURCE) with lens was chosen to capture the paddy field environment information in real time. A computer as a process unit, running on Windows 10 operation system, was used for real-time image processing. The specifications of the camera and computer are shown in Table 1. 

**Figure 1:**
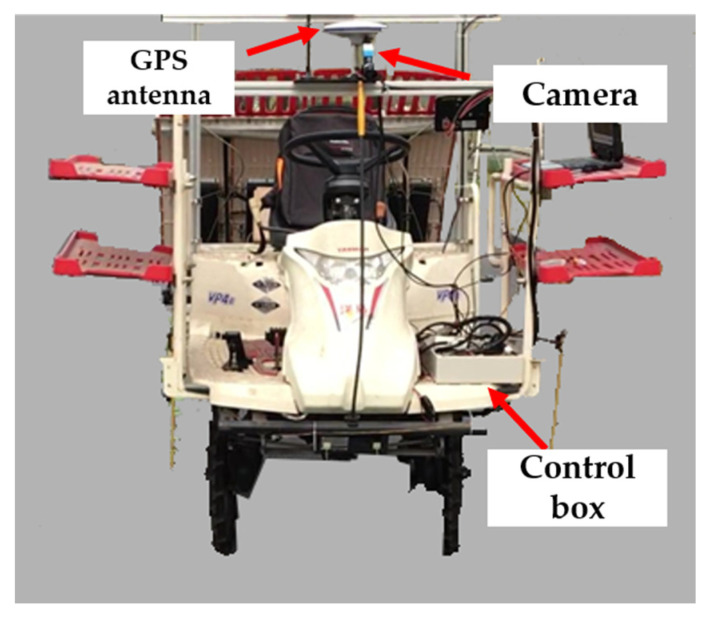
Transplanter platform and its machine vision system.

The camera was connected to the processing unit via USB 3.0 interface. In order to test the performance of this proposed machine-vision system, a remolded 4-transpanting-rows transplanter (VP4E, YANMAR) was used as an experimental platform which can realize autonomous navigation using the Global Positioning System (GPS). The camera was mounted on a crossbar in front of the center line of the transplanter, and the processing unit was fixed in an industrial control box. The connecting-used USB 3.0 data cable was tied to the steel frame to ensure the stability of the connection. The developed platform was shown in Figure 1.

### 2.2. Dataset

In the paddy field, obstacles could be divided into static obstacles such as electric poles, stone, window, etc., and moving obstacles such as large agricultural machines, people, livestock, etc. As for the known static obstacles, reasonable path planning in automatic navigation for intelligent agricultural machines could avoid those well. However, for moving obstacles, due to the uncertainty of their positions, the established planned path is more likely to hurt them. Thus, to avoid those moving obstacles, the machines have to detect and track them in real time to know where they are and update the control strategy. Humans are the first moving obstacle considered that must be carefully avoided. Because safety is the primary factor while working, the occurrence rate of an emergency should be minimized. In addition, water buffaloes are one of the common animal powers widely used in a paddy field in developing countries and areas. Thus, human and water buffaloes are selected as the moving obstacles to be detected and tracked in this research. 

There are some open source datasets for obstacle detection in agriculture like FieldSAFE [22]. FieldSAFE just contains humans in an agricultural environment, but not in paddy field. Because there is no open source standard dataset containing human and water buffaloes in paddy field, so it’s necessary to create a dataset for detection training. In this dataset, images are obtained through a web crawler by searching key words of ‘water buffaloes in paddy field’ and ‘farmers in paddy field’ on Google Images. To ensure the reliability of this dataset, authors deliberately select diverse images in which people are from different regions. Besides, images containing water buffaloes also consist of various kinds of water buffaloes. In addition, the farmers and water buffaloes in images are in different postures, which could help the deep-learning model to be more robust while detecting. The most important is that all the farmers and water buffaloes in images have legible feature details. The total number of captured images from Google Images is 499 (humans appear 312 times, water buffaloes appear 241 times). In order to make the deep learning model overfit slower, authors adopted data augmentation. In data augmentation progress, the 499 original images were randomly flipped horizontally or vertically. Hence, the dataset includes 998 images in total. 

**Figure 2:**
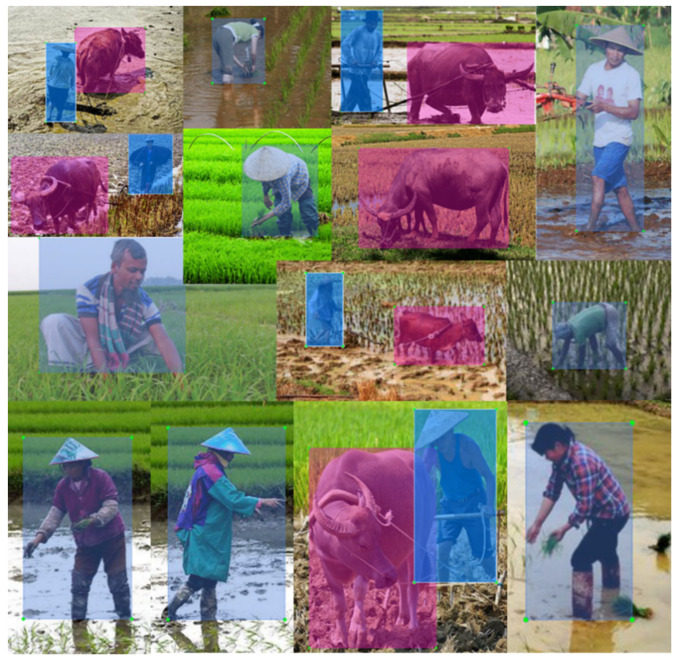
Parts of labeled images in dataset. The pink and blue rectangles are self-labeled ground truths. The pink rectangles are ground truths for water buffaloes, the blue ones are for humans.

After that, another indispensable operation is to label those images. In this research, the authors used an open source images label tool LabelImg (v1.8.3) to finish the label task. There are two classes of labels in this dataset, ‘person’ and ‘water buffalo’. The labelled dataset of persons and water buffaloes in paddy fields was saved as shown in Figure 2, where the blue or pink rectangles are ground truths. 

### 2.3. Image-Processing Methods

In order to recognize the moving obstacles in paddy fields (farmers and water buffaloes in this research), detecting the moving obstacles accurately and rapidly, tracking the objects and obtaining the coordination of their real-time position is an effective means. Authors adopted an approach to solve the detection and tracking tasks. To detect moving obstacles, a re-designed neural network based on Yolov3 was applied. Meanwhile, deep SORT [23] was a promising method to track the moving obstacles. The method framework was built by Python-3.7 mainly with the deep-learning framework Tensorflow-2.1.0 and the third part library opencv-3.4.0. 

#### 2.3.1. Object Detection Method

This research re-designed a new convolutional neural network (CNN) structure based on “Yolov3” as the objects detection method [24]. You Only Look Once (YOLO) series object detection methods are end-to-end methods with high processing speed [25,26]. Yolov3 method, which is the 3^rd^ version of Yolo method, segments an RGB image frame into N×N sized grids and processes it by a multi-scale prediction method similar to Feature Pyramid Networks (FPN) [27]. And the bounding boxes are predicted at 3 different scales, each box predicts the classes if it contains object using multi-label classification. Independent logistic classifiers are used to undertake class prediction instead of softmax. As for clustering, Yolov3 still uses k-means to determine the a prior bounding box, selects 9 clusters and 3 scales, and divides the 9 clusters evenly on 3 scales.

**Figure 3:**
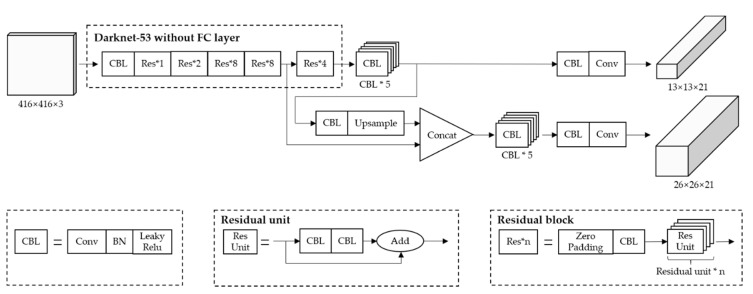
New improved Yolov3 network architecture.

Based on Yolov3, authors designed a more efficient neural network structure in this research to suit the presented application. The improved Yolov3 structure is shown in Figure 3. As for feature extracting tasks, this structure uses the open source framework “Darknet-53” which has 23 residual blocks. 

For predicting tasks, this structure predicts 3 boxes for every grid at 2 scales separately, and the predicted 3-d tensor is N×N×[3×(1+4+2)], which is encoded with 1 objectness prediction, 4 bounding boxes offsets, and 2 class predictions in this research. The feature map from 2 layers previous are upsampled by 2× then this structure concatenates it with other feature map from earlier layers. Compared to standard Yolov3, the improved Yolov3 just upsamples once which gives up smaller receptive fields to detect objects in images. Actually, in practical applications the speed of intelligent agriculture machines working in paddy field is not fast. There is no necessity to pursue an excessive accuracy when detecting the obstacles in the distance. Because they are far from the camera, those obstacles have few feature details. Moreover, detecting the obstacles in the distance would make efficiency loss and waste computing resources. Then those feature maps are processed by a few more 1 × 1, 3 × 3 convolutional layers to obtain detection results. 

Because of some changes in network structure, the original loss function would not be suitable in training. So, authors designed a new loss function for the improved Yolov3 network. The new loss calculation function is shown in Equation (1).

(1) $$L\left(t\_o,\;p\_o,\;t\_c,\;p\_c,t\_b,p\_b\right)=L_{conf}\left(t\_o,\;p\_o\right)+L_{class}\left(t\_c,\;p\_c\right)+L_{bbox}\left(t\_b,\;p\_b\right)$$

The loss calculation function in Equation (1) includes 3 part: confidence loss $L_{conf}\left(to,\;po\right)$, classification loss $L_{class}\left(tc,\;pc\right)$ and bounding box loss $L_{bbox}\left(t,\;p\right)$, which are shown in the equations below.

(2) $$L_{conf}\left(t\_o,\;\hat{p\_o}\right)=-\sum (t\_o_{i}\ln\left({\hat{p\_o}}_{i}\right)+\left(1-t\_o_{i}\right)\ln\left(1-{\hat{p\_o}}_{i}\right))$$

In the confidence loss calculation function, shown in Equation (2), t_o∈{0,1}, is a variable named true confidence. This variable shows whether there is an object in a bounding box, if true, t_o=1, if not, t_o=0; $\hat{p\_o}$ is a variable obtained from sigmoid(p_o) to indicate predicted confidence between 0 and 1.

(3) $$L_{class}\left(t\_c,\;\hat{p\_c}\right)=-\underset{i\in bbox}{\sum }\underset{j\in class}{\sum }(t\_c_{ij}\ln\left({\hat{p\_c}}_{ij}\right)+\left(1-t\_c_{ij}\right)\ln\left(1-{\hat{p\_c}}_{ij}\right))$$

Equation (3) is adopted to calculate classification loss, where $t\_c_{ij}$ is true classification score of the jth class in the ith bounding box and ${\hat{p\_c}}_{ij}$ is the predicted one. $\hat{p\_c}$ is also a variable between 0 and 1 from sigmoid(p_c) instead of softmax(p_c), so compared to traditional multi-label classification, $\sum_{j\in class}{\hat{p\_c}}_{j}$ is not equal to 1.

(4) $$L_{bbox}\left(t\_b,\;p\_b\right)=\underset{i\in bbox}{\sum }\underset{m\in \left\{x,y,w,h\right\}}{\sum }{\left(t\_b_{i}^{m}-p\_b_{i}^{m}\right)}^{2}$$

Bounding box loss is significant which is obtained by Equation (4). In Equation (4), the $t\_b_{i}$ contains 4 offsets of the ith bounding box as well as $p\_b_{i}$ contains predicted ones. These 4 offsets are center point positions x,y and weight w, height h of ground truth or bounding box. And the bounding boxes will be displayed in blue.

#### 2.3.2. Objects Tracking Method

In this research, “deep SORT (Simple Online and Realtime Tracking)” was chosen as the objects tracking method. The objects tracking method deep SORT is an online tracker with competitive performance to the state-of-the-art online trackers. It is a tracking-by-detection method, which defines the tracking scenario on an eight-dimensional state vector ($x,y,\gamma ,h,\dot{x},\dot{y},\dot{\gamma },\dot{h}$) that contains the bounding box center position (x,y) and height h from improved Yolov3 structure, aspect ratio γ and their respective velocities in image coordinates. The updated trajectory is predicted using a standard Kalman filter with constant velocity motion and linear observation model. The direct observations of the object state are bounding coordinates (x,y,γ,h). 

For each track there is a threshold $a_{k}$ for recording the time from the last successful match to the current time. When the value is greater than the threshold $A_{max}$ set in advance, the track is considered to be terminated. 

To solve the assignment problem, deep SORT provides two metrics of motion and appearance information. The squared Mahalanobis distance between predicted Kalman states and newly arrived measurements is used to incorporate motion information as shown in Equation (5):

(5) $$d^{\left(1\right)}\left(i,j\right)={\left(d_{j}-y_{i}\right)}^{T}S_{i}{}^{-1}\left(d_{j}-y_{i}\right)$$

where the projection of the i-th track distribution into measurement space is denoted by ($y_{i}$, $S_{i}$) and the j-th bounding box detection is denoted by $d_{j}$. 

The second metric shows the appearance information between the i-th track and j-th detection using the smallest cosine distance as shown in Equation (6):

(6) $$d^{\left(2\right)}\left(i,j\right)=min\left\{1-r_{j}{}^{T}r_{k}^{\left(i\right)}|r_{k}^{\left(i\right)}\in R_{i}\right\}$$

where the appearance descriptor $r_{j}$ is computed for each bounding box with $\|r_{j}\|=1$. In addition, a gallery $R_{k}={\left\{r_{k}^{\left(i\right)}\right\}}_{k=1}^{L_{k}}$ where the value $L_{k}$ indicates the last number of associated appearance descriptors for each track k is kept.

The combination using a weighted sum of both metrics is applied to solve the association problem as shown in Equation (7):

(7) $$c_{i,j}=\lambda d^{\left(1\right)}\left(i,j\right)+\left(1-\lambda \right)d^{\left(2\right)}\left(i,j\right)$$

Deep SORT adopts a cascading matching strategy, which allows the ’more frequently seen objects’ to be assigned a higher priority. In this way, the trajectory with the same occlusion time can be considered to allocate each time. In the end, deep SORT would display the white tracking boxes, an obstacle’s ID, and center point positions (x,y) if the obstacle is successfully tracked. 

**Figure 4:**
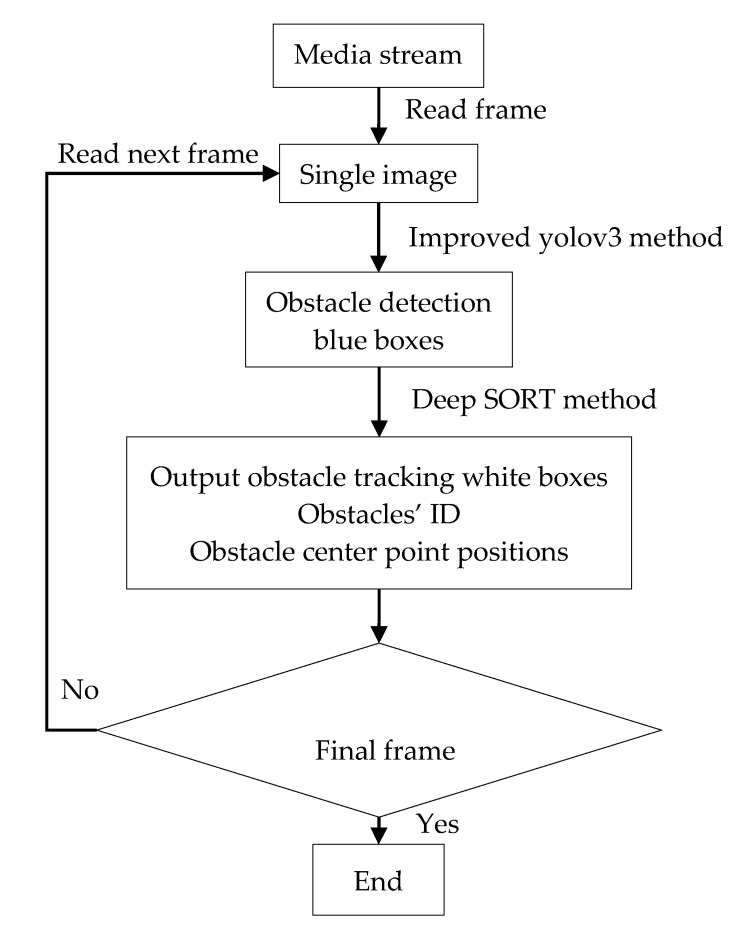
Flowchart of obstacle detecting and tracking.

The process flowchart is shown in Figure 4. This method reads frame from camera in real time and transmits the frame into the improved Yolov3 structure for detecting. After the detection method, some detection blue boxes would be generated and displayed. Then those blue boxes are sent to the deep SORT structure to figure out white tracking boxes, center point positions, and ID of the obstacle drawing them on the frame. This process would repeat until there is no next frame. 

## 3. Results and Discussion

### 3.1. Data Training

The improved Yolov3 structure applies anchor prior, so it is necessary to determine bounding box priors to be used in training. As mentioned above, this model would predict three 3-d tensors at two scales, so there are 6 anchors in total. These 6 anchors were obtained by clustering by k-means, which is a useful algorithm to cover it. In k-means, authors used Intersection over Union (IoU) to compute distance as a basis for clustering. On the self-created dataset, the 6 clusters are shown in Table 2. 

**Figure 5:**
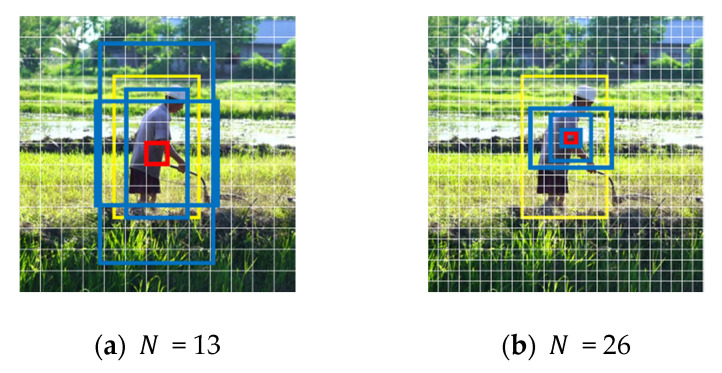
Sized grid at different scales. The blue boxes are the priori boxes obtained by clustering, the yellow one stands for ground truth, and the red one is the grid where the center point of object locates in.

These 6 clusters’ visual performance on the image is shown in Figure 5. The situation of N is 13, 26 separately at each scale in Figure 5a,b. 

After clustering, the obtained a priori boxes are the basis for training. The training process was carried out with the self-created dataset, 65% of which are randomly selected as the training set, and the remaining 35% are selected as the validation set. In order to improve the accuracy of this new model, authors decided to adopt transfer learning which requires a pre-trained model and pre-trained weights. The pre-trained weights file was downloaded from the Yolov3 official web, it was an open source file and generated through training on the Common Objects in Context (COCO) dataset [28]. 

**Figure 6:**
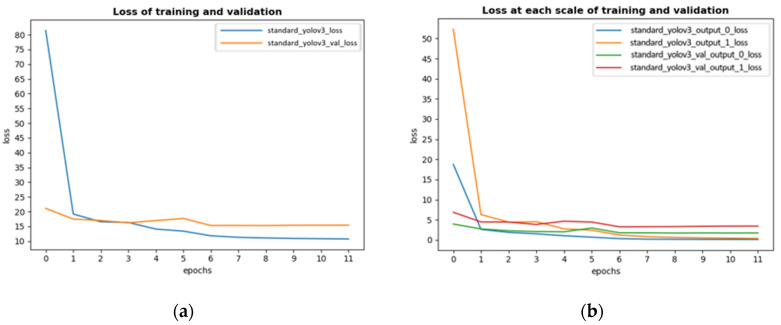
Convergence process of loss of improved Yolov3. (**a**) showed the training and validation loss of improved Yolov3. (**b**) showed the training and validation loss of improved Yolov3 at each scale. In Figure 6b, the ‘0’ in ‘output_0_loss’ means the loss at 13 × 13 scale; the ‘1’ in ‘output_1_loss’ means the loss at 26 × 26 scale.

**Figure 7:**
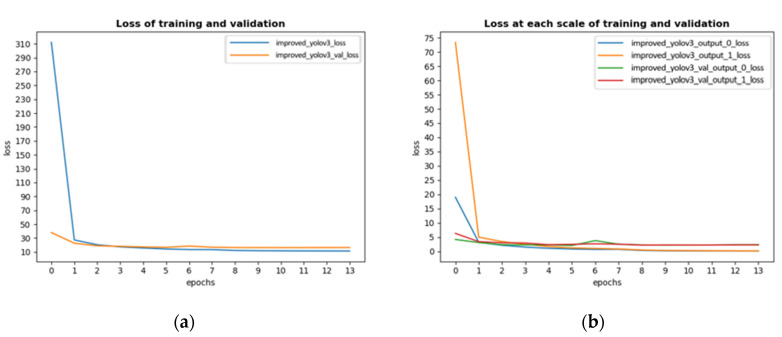
Convergence process of loss of standard Yolov3. (**a**) showed the training and validation loss of standard Yolov3. (**b**) showed the training and validation loss of standard Yolov3 at each scale. In Figure 7b, the ‘0’ in ‘output_0_loss’ means the loss at 13 × 13 scale; the ‘1’ in ‘output_1_loss’ means the loss at 26 × 26 scale.

In the training process, there were some parameters to adjust and the most important parameter was the learning rate, which was set as 0.001 at the beginning. Actually, a fixed learning rate would make the model oscillate when it converged, so the authors reduced the learning rate by half if validation loss did not decline in 2 epochs. To increase training efficiency, the authors adopted early stopping function. This function can stop training if validation loss did not decline in 3 epochs. The reason why validation loss was selected to be monitored is to avoid overfitting. The loss on train dataset and validation dataset of improved Yolov3 is shown in Figure 6 with optimizer of Adam and batch size of 2. As comparison, authors trained standard Yolov3 with the same parameters’ settings on the self-created dataset. The loss of standard Yolov3 is shown in Figure 7. 

The improved Yolov3 trained 12 epochs in total. Figure 6 showed the convergence process of training loss and validation loss. Figure 6a showed training loss and validation loss, because of early stopping function, this model stopped training after 12 epochs, which meant the minimum value of validation loss appeared at the end of the 9-th epoch. According to this figure, it was obvious that validation loss tended to be stable after 6 epochs and even increased after 9 epochs while training loss still went down, this phenomenon indicated that this model was over fitting; Figure 6b contained some interesting information: yolo_output loss was metric to measure the accuracy of prediction results at each scale. It could be clearly seen from this figure that output_0_loss converged faster because the receptive field was larger. 

Standard Yolov3 trained 14 epochs totally in the dataset. According to Figure 7, the convergence process is similar to that of the improved Yolov3. In the first few epochs of training, standard Yolov3 got higher loss, but had a rapid convergence later. The minimum value of validation loss appeared at the end of 11 epochs. 

Finally, the weights generated after 9 epochs were selected for improved Yolov3. This was because after 9 epochs, the improved Yolov3 was overfitting. In addition, the minimum value of validation loss appeared then. At each scale, the validation output loss was 1.70 and 3.29 respectively. And the weights file generated after 11 epochs was selected for standard Yolov3 for comparison. 

### 3.2. Validation on Internet Media 

**Figure 8:**
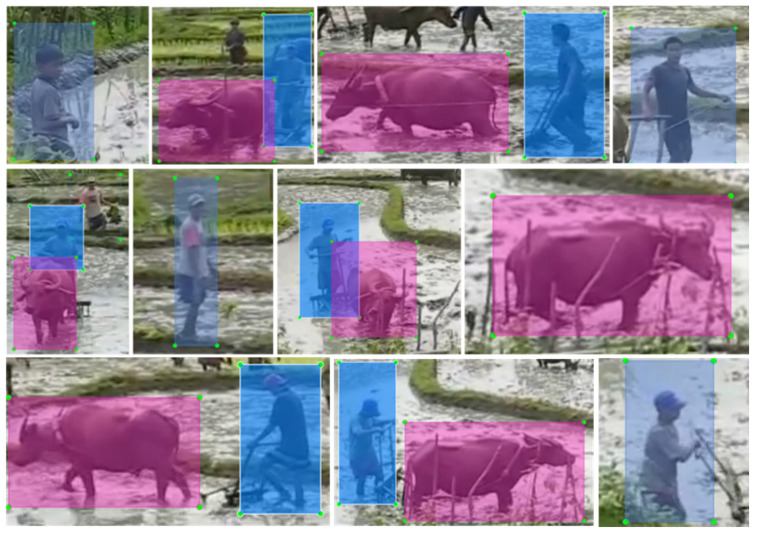
Parts of images in the test set. The pink and blue rectangles are self-labeled ground truths. The pink rectangles are ground truths for water buffaloes, the blue ones are for humans.

To verify the robustness of this proposed network, the authors made a test set. Those images in this dataset were taken from an online media containing the scene of a paddy field [29]. Authors selected 10 frames randomly from this media and cut them into 14 images. Parts of the labeled images by LabelImg (v1.8.3) are shown in Figure 8. 

According to Figure 8, images in the test set show the working environment under real conditions and had no connections with training or validation dataset. Therefore, running on this image set could reflect the performance of this improved Yolov3 structure to a certain degree with measurement of mean IoU (mIoU). 

To verify the performance of this improved Yolov3 structure, the authors compared it with standard Yolov3. The weights by which Yolov3 loaded were generated by training on self-created dataset under the same conditions as improved structure. Compared to standard Yolov3, this improved Yolov3 structure obtained almost the same score on the mIoU performance. Moreover, in terms of processing speed, the improved structure was 27.3% faster than standard Yolov3. Performance comparison of these two methods is shown in Table 3. 

### 3.3. Practical Application 

Testing on a dataset from online media is a useful way to measure the reliability of the developed structure. However, it was still theoretical. This structure needs to be applied in real paddy field conditions to evaluate the performance of this structure. The practical experiment was conducted in an experimental paddy field of Zhejiang Province (Xiaoshan) Modern Agriculture Innovation Park located in Hangzhou, China. Due to limitations, no water buffalo could be used. In the experiment, there were two farmers working in the paddy field environment. Various postures and phenomena were recorded by the camera. The chosen resolution used in practical application is 640 × 480, which is precise enough and highly-efficient.

**Figure 9:**
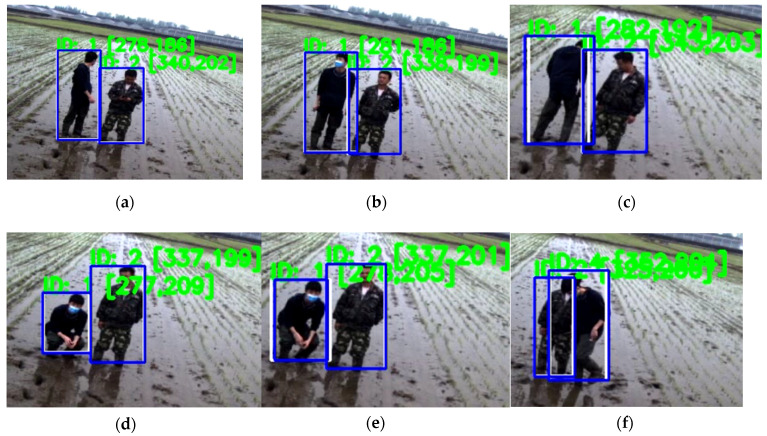
Performance of proposed method. The blue boxes are bounding boxes, the white boxes are tracking boxes. IDs of tracked farmers and center point position of each farmer are figured out by the green color.

In this experiment, detecting network-improved Yolov3 and tracking method deep SORT were combined. The parameters in deep SORT were default values, which were trained from the MOT16 dataset [30]. The performance of the detecting and tracking method is shown in Figure 9. 

Several typical scenes in paddy fields were recorded. In Figure 9a–c, the camera captured the side, front and back of the person on the left of the screen, who was accurately detected and tracked. In those 3 frames this person still kept standing, and according to the outputs, the proposed method could effectively detect and track standing person from several typical angles; in Figure 9d, the person on the left of screen was squatting, and was still detected and tracked; in Figure 9e, the same person was bent over, who could also be detected and tracked. From these 5 images, it could be concluded that for detecting and tracking this proposed method was competent and could be applied into real conditions. 

Overlap phenomenon between people is very common during work as shown in Figure 9f. It is meaningful and a prerequisite to handle the detection and tracking tasks under this condition. In Figure 9f, the two overlapping persons were successfully detected and tracked. It showed the capability of this method to process the overlap problem. 

**Figure 10:**
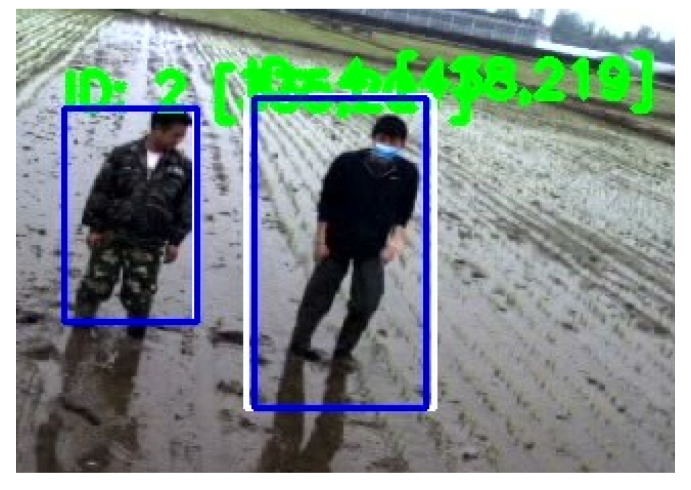
Detection error of the proposed method. The blue boxes are bounding boxes, the white boxes are tracking boxes. IDs of Tracked farmers and center point position of each farmer are figured out by the green color.

However, there was still a detection error deserving attention in this experiment as shown in Figure 10. Figure 10 shows the localization error of the proposed method. For the farmer on the left side, the blue box was relatively small. His head and left leg were partially out of the blue bounding box; For the farmer on the right side, the blue bounding box was relatively large. The cause of this localization error reasonably was the water environment of the paddy field. Water reflection is a major noise to influence the accuracy of the object detection method. In future, samples in training dataset should be enriched to make this approach more robust. Some image pre-processing methods should also be attempted to eliminate the noise of water reflection. 

In summary, this proposed method could successfully detect and track people shot from various angles at work in paddy fields, whether the person was standing, bent over or squatting. The process speed was 5–7 FPS by the used computing power in this research. 

## 4. Conclusions

Detecting and tracking moving obstacles is the basis of autonomous navigation for intelligent agricultural machines working in paddy fields. In this regard, this paper presented a method using machine vision with deep-learning methods to detect and track human and water buffaloes in paddy field environment. For detecting moving obstacles, an improved Yolov3 method was proposed, which has 23 residual blocks and predicts boxes at 2 scales. The loss function supports improved Yolov3, converges faster and makes better detection effects. Through a performance comparison of mIoU on an internet media, the improved Yolov3 has almost same detection ability and is 27.3% faster detection speed compared to the standard Yolov3; and the processing speed of the proposed method including detecting and tracking moving obstacles could reach an average 5–7 FPS on the processing unit in this research. This could guarantee real-time processing. 

An efficient and feasible obstacle avoidance method could effectively reduce the accident rate in automatic agriculture machine operations. In the further study, more samples from real-word scenarios should be included in the dataset to make the proposed approach more robust in order to minimize the effect of environmental factors. In addition, the training samples should be enriched with more classes of moving obstacles common in paddy fields to make the proposed method more suitable and adoptable for practical applications. 

In the future, the obtained center point positions of the obstacles by using this proposed method could be used to predict the movement trend of the obstacles, which could be combined with simultaneous localization and mapping (SLAM) or other technologies to know exactly where the moving obstacles are and how far away those obstacles are from the machine. This could be promising to support developing the obstacle avoidance system. With proposed method, those intelligent agricultural machines could detect and track the moving obstacles. In this way, the intelligent agricultural machines could decide what kinds of control strategies, like avoiding the obstacles or warning them to move aside, to be implemented. Finally, it is likely to realize a robust autonomous navigation system for intelligent agricultural machines working in paddy fields. 

## Figures and Tables

**Table 1:** Specifications of the camera and the processing unit.

Camera		Computer	
Sensitivity	0.05 lx	Central Processing Unit (CPU)	Intel Core^®^ i7-6700HQ
Video formats @ frame rate (maximum)	1280 × 960 (1.2 MP) RGB32 @ 30 fps 1280 × 960 (1.2 MP) Y800 @ 30 fps 1280 × 960 (1.2 MP) Y16 @ 30 fps	Graphic Processing Unit (GPU)	Nvidia GTX 970M
Temperature (operating)	−5 °C to 45 °C	Memory	16 GB 64-bit DDR4
Humidity (operating)	20 % to 80 % (non-condensing)	Universal Serial Bus (USB)	3x USB 3.0, USB 2.0 Micro-B

**Table 2:** Size of 6 clusters at each scale.

Scale (grid)	13 * 13	26 * 26
Clusters (Pixel)	157 × 183 193 × 92 331 × 171	25 × 29 69 × 61 89 × 123

**Table 3:** Performance comparison of standard Yolov3 and improved Yolov3 on test set.

	mIoU	FPS
Yolov3	0.791	11
Improved Yolov3	0.779	14
